# A Novel Grid SINS/DVL Integrated Navigation Algorithm for Marine Application

**DOI:** 10.3390/s18020364

**Published:** 2018-01-26

**Authors:** Yingyao Kang, Lin Zhao, Jianhua Cheng, Mouyan Wu, Xiaoliang Fan

**Affiliations:** College of Automation, Harbin Engineering University, Harbin 150001, China; kangyingyao@hrbeu.edu.cn (Y.K.); wumouyan@hrbeu.edu.cn (M.W.); fanxiaoliang@hrbeu.edu.cn (X.F.)

**Keywords:** middle-high latitude regions, grid frame, integrated navigation, unified Earth model, ARKF hybrid-correction

## Abstract

Integrated navigation algorithms under the grid frame have been proposed based on the Kalman filter (KF) to solve the problem of navigation in some special regions. However, in the existing study of grid strapdown inertial navigation system (SINS)/Doppler velocity log (DVL) integrated navigation algorithms, the Earth models of the filter dynamic model and the SINS mechanization are not unified. Besides, traditional integrated systems with the KF based correction scheme are susceptible to measurement errors, which would decrease the accuracy and robustness of the system. In this paper, an adaptive robust Kalman filter (ARKF) based hybrid-correction grid SINS/DVL integrated navigation algorithm is designed with the unified reference ellipsoid Earth model to improve the navigation accuracy in middle-high latitude regions for marine application. Firstly, to unify the Earth models, the mechanization of grid SINS is introduced and the error equations are derived based on the same reference ellipsoid Earth model. Then, a more accurate grid SINS/DVL filter model is designed according to the new error equations. Finally, a hybrid-correction scheme based on the ARKF is proposed to resist the effect of measurement errors. Simulation and experiment results show that, compared with the traditional algorithms, the proposed navigation algorithm can effectively improve the navigation performance in middle-high latitude regions by the unified Earth models and the ARKF based hybrid-correction scheme.

## 1. Introduction

More and more significant scientific research and shipping industry are carried out in the polar region [1]. No matter in or heading to the polar region, navigation plays an important role to ensure the safety and reliability of vehicles in marine application, so it is significant to ensure the navigation performance in middle-high latitude regions [2]. With the latitude increasing, the global navigation satellite system (GNSS) would suffer more and more challenges such as ionosphere scintillation, multipath effect and lack of reference stations [3,4]. Although the strapdown inertial navigation system (SINS) is highly autonomous and self-contained [5,6], the SINS navigation output contains three kinds of periodic oscillation errors and accumulated errors [7,8,9]. To restrain the SINS errors, the integrated navigation system of SINS and other external navigation information is usually applied. The Doppler velocity log (DVL) is a good acoustic-based device in marine applications, which can provide three-dimensional velocities to restrain the errors of marine SINS [10,11,12]. Therefore, the SINS/DVL integration is a potential method for highly accurate marine navigation when the vehicles are in or heading to the polar region.

The traditional SINS based on north-oriented geographic frame performs well in middle latitude regions but loses its effectiveness in high latitude regions because of the meridian convergence [13]. The wander frame is a traditional solution in high latitude regions, however it cannot provide position and orientation information near the pole [13]. A grid frame is proposed and the grid SINS algorithm is designed in [14], which can solve the problem of meridian convergence by setting an available reference line. In the traditional SINS, the north-oriented geographic frame is used in middle latitude regions and the grid frame is used in high latitude regions, but the errors will occur during the switch between these two frames. In fact, the grid SINS can also work in middle latitude regions in theory [14,15]. Therefore, if the grid SINS based integrated navigation algorithm is designed properly, the grid frame will be a reasonable choice for navigation system without navigation frame switch when working in middle-high latitude regions. In [15,16,17], the traditional north-oriented SINS based integrated navigation algorithms have been applied. However, when working in high latitude regions, the navigation calculation error caused by meridian convergence is one of main SINS error sources, which will not be estimated and restrained by the integrated filter. Thus, the traditional north-oriented integrated navigation systems cannot work properly in high latitude regions. The grid SINS based integrated navigation algorithms are proposed for flight and the GNSS and star tracker are employed as external measurements [18,19]. However, for marine application, the GNSS and star tracker cannot achieve the long-distance signal transmission under water. The acoustic-based DVL is a proper choice for marine application in integrated navigation systems. In [18,19], the integrated filter models are designed based on a sphere Earth model, while the SINS mechanizations are based on a reference ellipsoid Earth model. The disunity of the Earth models between the SINS mechanization and the integrated filter model will bring the principle errors and decrease the navigation accuracy, especially in middle latitude regions. Thus, it is necessary to choose a unified accurate Earth model and design an accurate grid SINS/DVL integrated filter model.

The accurate filter model, filter algorithm and correction scheme are important factors to ensure the navigation accuracy. The Kalman filter (KF) is a well-known filter method for integrated navigation applications [20]. However, the performance of KF depends on many factors such as filter models and noise characteristics [20,21]. Besides, the traditional KF is susceptible to measurement outliers [22,23]. It is noted that the uncertain measurement errors from acoustic-based sensors (such as DVL) are unavoidable, so an adaptive filter method is a proper choice for the SINS/DVL integrated system [24,25,26,27]. Finally, in traditional integrated navigation systems, the output-correction and feedback-correction are generally used as the correction schemes of the system, which will both impact the navigation system outcome through the correction. The output-correction integrated system cannot correct the SINS inner errors, so the accuracy of the filter would decrease sharply when the output-correction integrated system works for a long time. The feedback-correction system can correct the SINS inner errors at the update frequency of the integrated filter, but the filter errors will be also introduced into the SINS. In the traditional algorithms, the filter algorithms and correction schemes are researched independently. However, it would be better if the filter method and correction scheme are considered as a whole in the design to achieve a better navigation performance considering the characteristics of the DVL measurement errors.

The main contribution of this paper is to propose a novel marine grid SINS/DVL integrated navigation algorithm which can improve the performance of navigation system when vehicles working in or heading to the polar region. In this paper, the grid SINS mechanization with the reference ellipsoid Earth model is introduced firstly. Then, the gird SINS error equations are derived based on the SINS mechanization. According to the new grid SINS error equations, a dynamic filter model is designed and a modified accurate integrated navigation algorithm is proposed with the same Earth model. Besides, the DVL output errors are analyzed according to the DVL operating characteristic. Considering the DVL output errors, an adaptive robust Kalman filter (ARKF) is introduced, which can detect and reduce the influence of measurement errors by an adaptive factor. Then, a hybrid-correction scheme is designed based on the ARKF with a switching criterion and two correction channels. With the help of switching criterion, the proposed integrated navigation system can restrain navigation errors and improve the accuracy by choosing the proper correction channel. Finally, simulations and semi-physical experiments are conducted to validate the performance of the proposed integrated navigation algorithm.

## 2. Grid SINS Mechanization

The World Geodetic System 1984 (WGS-84) Earth model is widely used as the national geodetic reference system and is also chosen as the reference ellipsoid Earth model in this paper to design the grid SINS/DVL integrated navigation algorithm. The main frames used in this paper are the inertial frame (denoted as i), the Earth centered Earth fixed (ECEF) frame (denoted as e), the geographic frame (denoted as g), the grid frame (denoted as G) and the body frame (denoted as b).

### 2.1. Grid Frame

**Figure 1:**
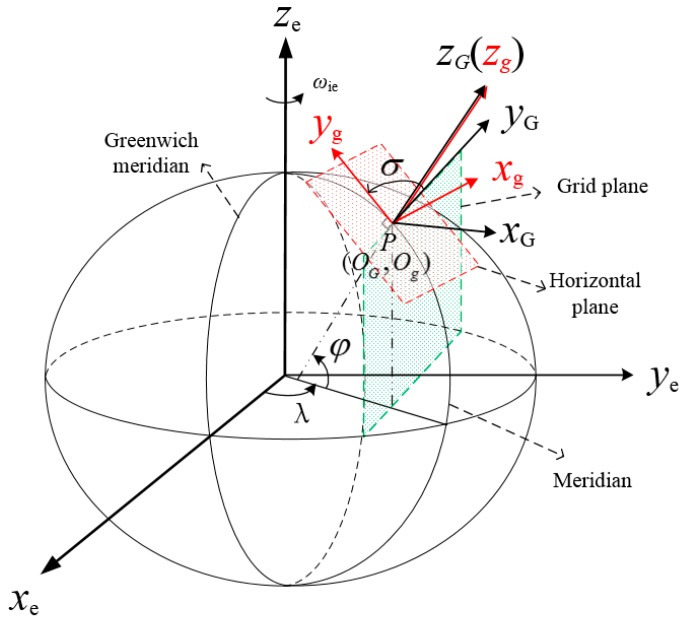
Description of the grid frame.

The grid frame is adopted to avoid the navigation errors caused by the meridian convergence. The mass center of the vehicle is chosen as the grid frame origin. As shown in Figure 1, when the mass center of the vehicle is located at point P, the origins of grid and geographic frames coincide with P; the local grid plane is the plane which passes through the point P and parallels to the Greenwich plane. The G frame is a right-handed coordinate system, and the axes are defined as follows.

The $oy_{G}$ axis is the grid north axis lying along the intersecting line of grid plane and local horizontal plane. The $ox_{G}$ axis is the grid East axis lying in the local horizontal plane and perpendicular to grid north axis. The $oz_{G}$ axis is the grid up axis perpendicular to the local horizontal plane and along the geographic frame *z*-axis.

The angle between the geographic north and grid north axis is σ. The latitude and longitude of point P are defined as φ and λ, respectively. The transformation matrix between e, g and G frames can be described as:

(1) $$C_{e}^{g}=\;\left[\begin{array}{ccc} -\sin\lambda & \cos\lambda & 0\\ -\sin\varphi \cos\lambda & -\sin\varphi \sin\lambda & \cos\varphi \\ \cos\varphi \cos\lambda & \cos\varphi \sin\lambda & \sin\varphi \end{array}\right]$$

(2) $$C_{g}^{G}=\;\left[\begin{array}{ccc} \cos\sigma & -\sin\sigma & 0\\ \sin\sigma & \cos\sigma & 0\\ 0 & 0 & 1 \end{array}\right]$$

The σ can be obtained by:

(3) $$\sin\sigma =\sin\lambda \sin\varphi /\sqrt{1-\cos^{2}\varphi \sin^{2}\lambda }$$

(4) $$\cos\sigma =\cos\lambda /\sqrt{1-\cos^{2}\varphi \sin^{2}\lambda }$$

### 2.2. Grid SINS Mechanization

The differential equations of SINS attitude, velocity and position in G frame are

(5) $${\dot{C}}_{b}^{G}=C_{b}^{G}(\omega_{Gb}^{b}\times )$$

(6) $${\dot{V}}^{G}=C_{b}^{G}f^{b}-\left(2\omega_{ie}^{G}+\omega_{eG}^{G}\right)\times V^{G}+g^{G}$$

(7) $${\dot{R}}^{e}=C_{G}^{e}V^{G}=\;{\left(C_{e}^{G}\right)}^{T}{\left(C_{G}^{G}\right)}^{T}V^{G}$$

where $C_{b}^{G}$ represents the calculated attitude matrix of SINS from b to G frame; $\omega_{Gb}^{b}$ is the angular velocity of G frame which is relative to b frame and is expressed in b frame; $\left(\omega_{Gb}^{b}\times \right)$ is the anti-symmetric matrix of $\omega_{Gb}^{b}$; $V^{G}$ is the velocity of vehicles expressed in G frame; $f^{b}$ is the specific force measured by SINS accelerometers; $\omega_{ie}^{G}$ is the angular velocity of Earth rotation expressed in G frame; $\omega_{eG}^{G}$ is the angular velocity of e frame which is relative to G frame and is expressed in G frame; $g^{G}$ is the projection of local gravity acceleration expressed in G frame; $R^{e}$ is the position coordinates expressed in e frame; and $C_{G}^{e}$ is the transformation matrix from G to e frame. $\omega_{Gb}^{b}$ can be obtained by:

(8) $$\omega_{Gb}^{b}=\;\omega_{ib}^{b}-C_{G}^{b}\omega_{iG}^{G}$$

(9) $$\omega_{iG}^{G}=\;\omega_{ie}^{G}+\;\omega_{eG}^{G}$$

(10) $$\omega_{ie}^{G}\;=\;C_{g}^{G}\omega_{ie}^{g}$$

where $\omega_{ib}^{b}$ is the output vector of SINS gyroscopes and $\omega_{ie}^{g}$ is the angular velocity vector of Earth expressed in g frame.

The parameter κ is defined as:

(11) $$\kappa =\frac{\sin\lambda \cos\varphi }{\sqrt{1-\cos^{2}\varphi \sin^{2}\lambda }}$$

Then, $\omega_{eG}^{G}$ can be described as:

(12) $$\omega_{eG}^{G}=\left[\begin{array}{c} \omega_{eGx}^{G}\\ \omega_{eGy}^{G}\\ \omega_{eGz}^{G} \end{array}\right]=\left[\begin{array}{cc} \frac{1}{\tau_{f}} & -\frac{1}{R_{y}}\\ \frac{1}{R_{x}} & -\frac{1}{\tau_{f}}\\ \frac{\kappa }{\tau_{f}} & -\frac{\kappa }{R_{y}} \end{array}\right]\left[\begin{array}{c} v_{E}^{G}\\ v_{N}^{G} \end{array}\right]$$

where ${R_{x}}^{-1}$ is the local Earth curvature along the grid East axis. ${R_{y}}^{-1}$ is the local Earth curvature along the grid north axis. ${\tau_{f}}^{-1}$ is the local Earth twist rate. They can be described as:

(13) $$\left\{ \begin{array}{l} \frac{1}{R_{x}}=\frac{\sin^{2}\sigma }{R_{Mh}}+\frac{\cos^{2}\sigma }{R_{Nh}}\\ \frac{1}{R_{y}}=\frac{\cos^{2}\sigma }{R_{Mh}}+\frac{\sin^{2}\sigma }{R_{Nh}}\\ \frac{1}{\tau_{f}}=\left(\frac{1}{R_{Mh}}-\frac{1}{R_{Nh}}\right)\sin\sigma \cos\sigma \end{array}\right.$$

where $R_{Mh}$ and $R_{Nh}$ are the local radiuses of Earth in the meridian circle and the prime vertical, respectively.

Because G frame has a local horizontal plane, $g^{G}$ can be described as:

(14) $$g^{G}={\left[\begin{array}{ccc} 0 & 0 & -g \end{array}\right]}^{T}$$

where g is the gravitational acceleration.

The position transformation from geodetic latitude, longitude, and altitude to ECEF coordinate is described as:

(15) $$\left\{ \begin{array}{l} x=R_{Nh}\cos\varphi \cos\lambda \\ y=R_{Nh}\cos\varphi \sin\lambda \\ z=[R_{N}(1-e^{2})+h]\sin\varphi \end{array}\right.$$

**Figure 2:**
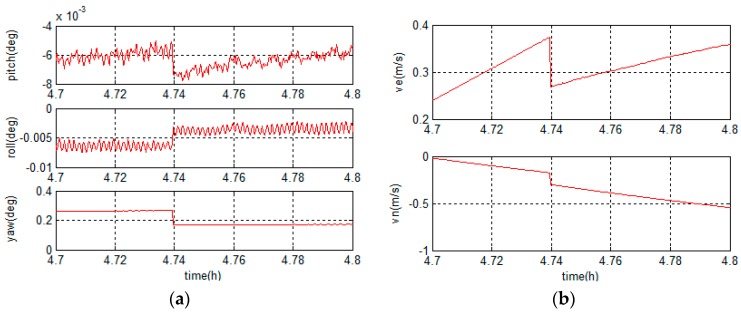
Navigation curves when navigation frame switches: (**a**) attitude curves; and (**b**) velocity curves.

The existing studies choose the north-oriented geographic frame as the navigation frame in middle latitude regions and choose the grid frame in high latitude regions. When vehicles navigate from middle latitude regions to high latitude regions, the switch of navigation frame is simulated and the attitude and velocity curves at the switch instant are shown in Figure 2.

As shown in Figure 2, the navigation curves jump when navigation frame switch occurs. Because the frame switch is a non-successive change, when a frame switch happens, some sudden changes will occur in the navigation system. To ensure the navigation accuracy when vehicles work in middle-high latitude regions, it is necessary to unify the navigation frame and improve the performance of grid frame based navigation system.

## 3. Error Equations of Grid SINS and DVL

In this section, new grid SINS error models are established and the error equations of DVL are derived. In the grid SINS, the main error sources are the gyroscope drifts (denoted as $\delta \omega_{ib}^{b}$) and the accelerometer biases (denoted as $\delta f^{b}$). The attitude velocity and position errors are denoted as $\phi ={\left[\begin{array}{ccc} \phi_{x} & \phi_{y} & \phi_{z} \end{array}\right]}^{T}$ $\delta V^{G}={\left[\begin{array}{ccc} \delta V_{x}^{G} & \delta V_{y}^{G} & \delta V_{z}^{G} \end{array}\right]}^{T}$ and $\delta P={\left[\begin{array}{ccc} \delta \varphi & \delta \lambda & \delta h \end{array}\right]}^{T}$, respectively. Because the grid SINS calculates position in ECEF coordinate, the position error in ECEF can be denoted as $\delta R^{e}={\left[\begin{array}{ccc} \delta x & \delta y & \delta z \end{array}\right]}^{T}$.

### 3.1. Earth Models and Parameters

It is known that the Earth is an irregular sphere and presents as an oval shape. In the navigation system, the Earth model should be described with special equations. There are three common Earth models in navigation, i.e., the geoid sphere, the reference ellipsoid and the sphere model. The geoid sphere is the most accurate one among the three models, but it is hard to be expressed by equations. The reference ellipsoid is close to geoid sphere, the deflections of the normal direction are less than $3^{\prime \prime }$ and the errors of radius are less than 150 m. The sphere is the most imprecise of the three models, and is normally used in theoretical analysis. Therefore, the reference ellipsoid Earth model is the common one employed in the practical integrated navigation.

**Figure 3:**
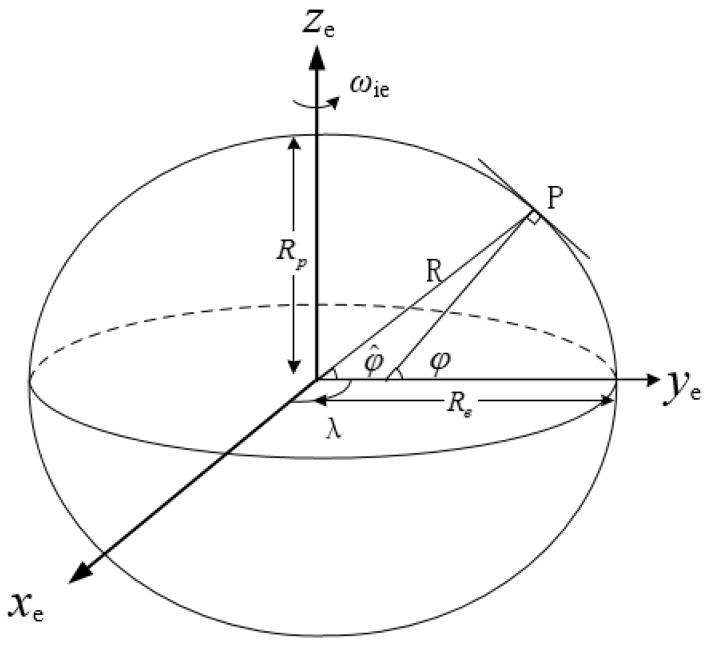
Description of the reference ellipsoid Earth model.

In the traditional grid SINS/DVL integrated navigation system, the sphere and the reference ellipsoid are both used as Earth models. When the Earth is modeled as a reference ellipsoid, the normal of reference ellipsoid model is called as the geographic vertical, and the geographic latitude φ is defined as the angle between geographic vertical and equatorial plane. When the Earth is modeled as a sphere, the connecting line between point P and Earth center is called the geocentric vertical, and the geocentric latitude $\hat{\varphi }$ is the angle between geocentric vertical and equatorial plane. The reference ellipsoid Earth model and two kinds of latitudes are shown in Figure 3. $R_{e}$ and $R_{p}$ are the two semi-axes of the Earth.

In SINS, the latitude is defined as geographic latitude. If the point P on the Earth is expressed by geographic and geocentric latitude, respectively, the latitude error caused by the disunity of the Earth models is defined as $\Delta \varphi =\varphi -\hat{\varphi }$, which can be described as:

(16) Δφ≈esin2φ

The maximum deviation between geographic and geocentric latitude occurs in middle-latitude regions, which can be up to $11^{\prime }$. The latitude error will bring decrease of the integrated navigation system accuracy especially in middle latitude regions. To improve the integrated navigation accuracy, it is necessary to unify the Earth models of the SINS mechanization and the integrated filter model.

In the grid SINS based on the reference ellipsoid Earth model, the local curvatures and local twist rate of Earth are described as $\frac{1}{R_{x}^{c}}=\frac{1}{R_{x}}+\delta \frac{1}{R_{x}}$, $\frac{1}{R_{y}^{c}}=\frac{1}{R_{y}}+\delta \frac{1}{R_{y}}$ and $\frac{1}{\tau_{f}^{c}}=\frac{1}{\tau_{f}}+\delta \frac{1}{\tau_{f}}$. Neglecting the second order small quantity and according to Equation (13), the errors of Earth curvatures and twist rate in G frame can be obtained as:

(17) $$\left\{ \begin{array}{l} \delta \frac{1}{R_{x}}=\;2\sin\sigma \cos\sigma \left(\frac{1}{R_{Mh}}-\frac{1}{R_{Nh}}\right)\delta \sigma \\ \delta \frac{1}{R_{y}}=-2\sin\sigma \cos\sigma \left(\frac{1}{R_{Mh}}-\frac{1}{R_{Nh}}\right)\delta \sigma \\ \delta \frac{1}{\tau_{f}}=-\left(\frac{1}{R_{Mh}}-\frac{1}{R_{Nh}}\right)(\sin^{2}\sigma -\cos^{2}\sigma )\delta \sigma \end{array}\right.$$

Deriving the first order differential of Equation (4), δσ can be obtained by:

(18) $$\delta \sigma \;=\;\left[\begin{array}{ccc} \frac{\sin\varphi }{1-\cos^{2}\varphi \sin^{2}\lambda } & \frac{\sin\lambda \cos\lambda \cos\varphi }{1-\cos^{2}\varphi \sin^{2}\lambda } & 0 \end{array}\right]\left[\begin{array}{c} \delta \varphi \\ \delta \lambda \\ \delta h \end{array}\right]\;=\;C_{1}\delta P$$

Deriving the first order differential of Equation (15), δP can be calculated as:

(19) $$\delta P=\;\frac{1}{R_{Nh}}\left[\begin{array}{ccc} -\sin\varphi \cos\lambda & -\sin\varphi \sin\lambda & \cos\varphi \\ -\frac{\sin\lambda }{\cos\varphi } & \frac{\cos\lambda }{\cos\varphi } & 0\\ R_{Nh}\cos\varphi \cos\lambda & R_{Nh}\cos\varphi \sin\lambda & R_{Nh}\sin\varphi \end{array}\right]\left[\begin{array}{c} \delta x\\ \delta y\\ \delta z \end{array}\right]\;=\;C_{R2P}\delta R^{e}$$

Then, the δσ can be obtained by Equations (18) and (19):

(20) $$\delta \sigma \;=\;C_{1}C_{R2P}\delta R^{e}\;=\;C_{R2\sigma }\delta R^{e}$$

According to Equations (17) and (20), the error equations of the Earth curvatures and twist rate in G frame can be calculated as:

(21) $$\left\{ \begin{array}{l} \delta \frac{1}{R_{x}}\;=\;2\sin\sigma \cos\sigma \left(\frac{1}{R_{Mh}}-\frac{1}{R_{Nh}}\right)C_{R2\sigma }\delta R^{e}\\ \delta \frac{1}{R_{y}}\;=\;-2\sin\sigma \cos\sigma \left(\frac{1}{R_{Mh}}-\frac{1}{R_{Nh}}\right)C_{R2\sigma }\delta R^{e}\\ \delta \frac{1}{\tau_{f}}\;=\;-\left(\frac{1}{R_{Mh}}-\frac{1}{R_{Nh}}\right)(\sin^{2}\sigma -\cos^{2}\sigma )C_{R2\sigma }\delta R^{e} \end{array}\right.$$

### 3.2. $\omega_{eG}^{G}$ Error Equations

According to the grid SINS mechanization, the accuracy of $\omega_{eG}^{G}$ will also be influenced by Earth model. When the Earth is considered as a sphere, then $1/R_{x}=1/R_{eh}$, $1/R_{y}=1/R_{eh}$, and $1/\tau_{f}^{c}=0$. $R_{eh}$ is the local radius of Earth. According to Equation (12), the error of $\omega_{eG}^{G}$ can be described as:

(22) $$\begin{array}{cc} \delta \omega_{eG\_ball}^{G} & =\;\omega_{eG\_ball}^{G^{c}}-\omega_{eG\_ball}^{G}\\ & =\left[\begin{array}{c} -\frac{1}{R_{eh}}\delta v_{G_{N}}\\ \frac{1}{R_{eh}}\delta v_{G_{E}}\\ -\frac{1}{R_{eh}}\kappa \delta v_{G_{N}}+\frac{v_{G_{N}}}{R_{eh}}\frac{\kappa }{\cot\varphi }\delta \varphi -\frac{v_{G_{N}}}{R_{eh}}\kappa \delta \sigma \end{array}\right] \end{array}$$

When the Earth is considered as a reference ellipsoid, according to the Equations (12) and (21), the error of $\omega_{eG}^{G}$ can be obtained by:

(23) $$\begin{array}{cc} \delta \omega_{eG}^{G} & =\;\omega_{eG}^{G^{c}}-\omega_{eG}^{G}\\ & =\left[\begin{array}{c} -\frac{1}{R_{y}}\delta v_{G_{N}}\\ \frac{1}{R_{x}}\delta v_{G_{E}}\\ -\frac{1}{R_{y}}\kappa \delta v_{G_{N}}+\left(\frac{v_{G_{N}}}{R_{y}}-\frac{v_{G_{E}}}{\tau_{f}}\right)\frac{\kappa }{\cot\varphi }\delta \varphi -\left(\frac{v_{G_{N}}}{R_{y}}-\frac{v_{G_{E}}}{\tau_{f}}\right)\kappa \cot\sigma \delta \sigma \end{array}\right]\;+\;\left[\begin{array}{c} \frac{1}{\tau_{f}}\delta v_{G_{E}}+v_{G_{E}}\delta \frac{1}{\tau_{f}}-v_{G_{N}}\delta \frac{1}{R_{y}}\\ -\frac{1}{\tau_{f}}\delta v_{G_{N}}-v_{G_{N}}\delta \frac{1}{\tau_{f}}+v_{G_{E}}\delta \frac{1}{R_{x}}\\ \frac{1}{\tau_{f}}\kappa \delta v_{G_{E}}+v_{G_{E}}\kappa \delta \frac{1}{\tau_{f}}-v_{G_{N}}\kappa \delta \frac{1}{R_{y}} \end{array}\right] \end{array}$$

It can be seen that the $\delta \omega_{eG}^{G}$ equation has advantages compared to the $\delta \omega_{eG\_ball}^{G}$ equation. The influence from local Earth parameters and Earth parameter errors is considered and the influence from velocity and velocity errors is also presented more comprehensively in the $\delta \omega_{eG}^{G}$ equation than in the $\delta \omega_{eG\_ball}^{G}$ equation. In this case, the error of $\omega_{eG}^{G}$ can be described more accurately by Equation (23), and Equation (23) will provide a better accuracy when it is used in the grid SINS/DVL integrated navigation system.

### 3.3. Grid SINS Attitude Error Equation

Quaternion algorithm is usually used in the implementation of attitude update; the quaternion error differential equation in grid frame can be described as:

(24) $$\delta \dot{Q}=-\frac{1}{2}\delta {\tilde{\omega }}_{ib}^{G}⊗\delta Q-\frac{1}{2}(\omega_{ie}^{G}+\omega_{eG}^{G})⊗\delta Q\;+\frac{1}{2}\delta Q⊗(\omega_{ie}^{G}+\omega_{eG}^{G}+\delta \omega_{ie}^{G}+\delta \omega_{eG}^{G})$$

where Q is the quaternion. The attitude error $\phi ={\left[\begin{array}{ccc} \phi_{x} & \phi_{y} & \phi_{z} \end{array}\right]}^{T}$ is considered as a small angle vector, and the quaternion Q can be described as:

(25) $$\delta Q=1+\frac{\phi }{2}$$

(26) $$\delta \dot{Q}=1+\frac{\dot{\phi }}{2}$$

According to Equations (25) and (26), and neglecting the second order small quantity, Equation (24) can be written as:

(27) $$\dot{\phi }=\phi \times \omega_{iG}^{G}+\delta \omega_{ie}^{G}+\delta \omega_{eG}^{G}-\delta {\tilde{\omega }}_{ib}^{G}$$

Besides, Equation (23) can be rewritten as:

(28) $$\delta \omega_{eG}^{G}\;=\;C_{2}\delta P+C_{3}\delta V+C_{4}\delta \sigma$$

where $C_{2}$, $C_{3}$ and $C_{4}$ can be expressed as:

(29) $$C_{2}=\;\left[\begin{array}{ccc} 0 & 0 & 0\\ 0 & 0 & 0\\ \left(\frac{v_{G_{N}}}{R_{y}}-\frac{v_{G_{E}}}{\tau_{f}}\right)\frac{\kappa }{\cot\varphi } & 0 & 0 \end{array}\right]$$

(30) $$C_{3}=\;\left[\begin{array}{ccc} \frac{1}{\tau_{f}} & -\frac{1}{R_{y}} & 0\\ \frac{1}{R_{x}} & -\frac{1}{\tau_{f}} & 0\\ \frac{\kappa }{\tau_{f}} & -\frac{\kappa }{R_{y}} & 0 \end{array}\right]$$

(31) $$C_{4}=\;\left[\begin{array}{c} v_{G_{N}}2\sin\sigma \cos\sigma \left(\frac{1}{R_{M}}-\frac{1}{R_{N}}\right)\\ v_{G_{E}}2\sin\sigma \cos\sigma \left(\frac{1}{R_{M}}-\frac{1}{R_{N}}\right)\\ v_{G_{N}}\kappa 2\sin\sigma \cos\sigma \left(\frac{1}{R_{M}}-\frac{1}{R_{N}}\right) \end{array}\right]\;+\;\left[\begin{array}{c} -v_{G_{E}}\left(\frac{1}{R_{M}}-\frac{1}{R_{N}}\right)(\sin^{2}\sigma -\cos^{2}\sigma )\\ v_{G_{N}}\left(\frac{1}{R_{M}}-\frac{1}{R_{N}}\right)(\sin^{2}\sigma -\cos^{2}\sigma )\\ -v_{G_{E}}\kappa \left(\frac{1}{R_{M}}-\frac{1}{R_{N}}\right)(\sin^{2}\sigma -\cos^{2}\sigma )-\left(\frac{v_{G_{N}}}{R_{y}}-\frac{v_{G_{E}}}{\tau_{f}}\right)\kappa \cot\sigma \end{array}\right]$$

According to Equation (10), the angular velocity error of Earth $\delta \omega_{ie}^{G}$ can be obtained by:

(32) $$\begin{array}{l} \delta \omega_{ie}^{G}=\omega_{ie}^{G^{c}}-\omega_{ie}^{G}\\ =\omega_{ie}\left[\begin{array}{c} -\cos\varphi \cos\sigma \\ -\cos\varphi \sin\sigma \\ 0 \end{array}\right]\delta \sigma \;+\;\omega_{ie}\left[\begin{array}{ccc} \sin\varphi \sin\sigma & 0 & 0\\ -\sin\varphi \cos\sigma & 0 & 0\\ \cos\varphi & 0 & 0 \end{array}\right]\left[\begin{array}{c} \delta \varphi \\ \delta \phi \\ \delta h \end{array}\right]\;=\;C_{5}\delta \sigma \;+\;C_{6}\delta P \end{array}$$

The attitude error equation can be calculated by Equations (27), (28) and (32):

(33) $$\dot{\phi }=-\omega_{iG}^{G}\times \phi +C_{V\_\phi }\delta V+C_{R\_\phi }\delta R^{e}-\delta {\tilde{\omega }}_{ib}^{G}$$

where $C_{V\_\phi }=C_{3}$ and $C_{R\_\phi }=(C_{6}+\;C_{2})C_{R2P}+(C_{5}+\;C_{4})C_{R2\sigma }$.

### 3.4. Grid SINS Velocity Error Equation

Based on the G frame, the actual velocity differential equation can be described as:

(34) $$\begin{array}{cc} {\dot{\hat{V}}}^{G} & ={\hat{C}}_{b}^{G}{\tilde{f}}^{b}-\left(2{\hat{\omega }}_{ie}^{G}+{\hat{\omega }}_{eG}^{G}\right)\times {\hat{V}}^{G}+{\hat{g}}^{G}\\ & =\;C_{G}^{G^{c}}C_{b}^{G}(f^{b}+\delta f^{b})+(g^{G}+\delta g^{G})-\left(2(\omega_{ie}^{G}+\delta \omega_{ie}^{G})+(\omega_{eG}^{G}+\delta \omega_{eG}^{G}\right)\times (V^{G}+\delta V^{G}) \end{array}$$

According to Equations (6), (28), (32) and (34), neglecting the second order small quantity and error of local gravity acceleration, the velocity error can be calculated as:

(35) $$\delta {\dot{V}}^{G}=\;f^{G}\times \phi +C_{V\_V}\delta V^{G}+C_{R\_V}\delta R^{e}+C_{b}^{G}\delta f^{b}$$

where $C_{R\_V}=\;\;\;(V^{G}\times )(2C_{6}+C_{2})C_{R2P}+(V^{G}\times )(2C_{5}+C_{4})C_{R2\sigma }$ and $C_{V\_V}=\;(V^{G}\times )C_{3}-(2\omega_{ie}^{G}+\omega_{eG}^{G})\times$.

### 3.5. Grid SINS Position Error Equation

Based on the ECEF frame, the actual position differential equation of the grid SINS can be written as:

(36) $${\dot{\hat{R}}}^{e}=C_{G^{C}}^{e}{\hat{V}}^{G}=C_{G}^{e}(I+\delta \theta^{G})(V^{G}+\delta V^{G})$$

where $\delta \theta^{G}$ is the error angle between G and $G^{c}$ frames. 

According to Equations (7) and (36), the position error can be calculated as:

(37) $$\delta {\dot{R}}^{e}=C_{G}^{e}\delta V^{G}-C_{G}^{e}(V^{G}\times )\delta \theta^{G}$$

**Figure 4:**
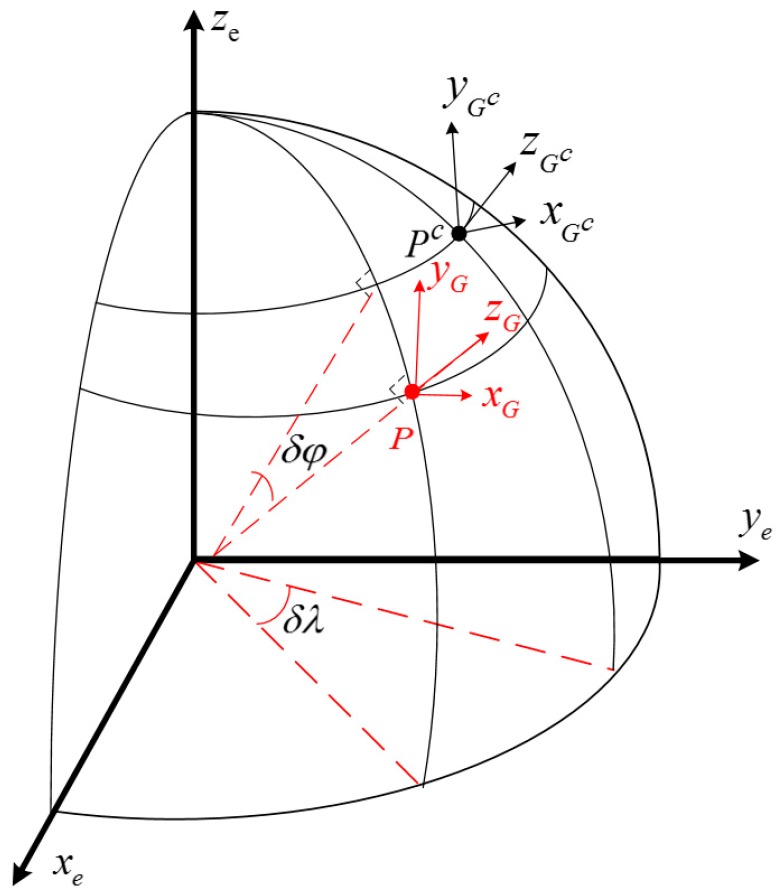
Relationship between G and $G^{c}$ frames.

As shown in Figure 4, when a vehicle is located at point P, the local grid frame is denoted as $Px_{G}y_{G}z_{G}$; the true values of latitude, longitude and angle between the true north and grid north are denoted as φ, λ and σ, respectively; the calculated grid frame is denoted as $P^{c}x_{G^{c}}y_{G^{c}}z_{G^{c}}$ and the calculated values are denoted as $\varphi^{c}$, $\lambda^{c}$ and $\sigma^{c}$, respectively.

The local G frame can be obtained by a three-time rotation from $G^{c}$ frame. The error $\delta \theta^{G}$ can be presented as:

(38) $$\begin{array}{cc} \delta \theta^{G} & =\;\left[\begin{array}{ccc} -\cos\sigma & -\cos\varphi \sin\sigma & 0\\ -\sin\sigma & \cos\varphi \cos\sigma & 0\\ 0 & \sin\varphi & 0 \end{array}\right]\left[\begin{array}{c} \delta \varphi \\ \delta \lambda \\ \delta h \end{array}\right]+\left[\begin{array}{c} 0\\ 0\\ -1 \end{array}\right]\delta \sigma \\ & =C_{7}\delta P+C_{8}\delta \sigma =(C_{7}C_{R2P}+C_{8}C_{R2\sigma })\delta R^{e}=\;C_{R2\delta \theta }\delta R^{e} \end{array}$$

According to Equation (38), Equation (37) can be rewritten as:

(39) $$\delta {\dot{R}}^{e}\;=\;C_{V\_R}\delta V^{G}+C_{R\_R}\delta R^{e}$$

where $C_{V\_R}=\;C_{G}^{e}$ and $C_{R\_R}=-C_{G}^{e}(V^{G}\times )C_{R2\delta \theta }$.

### 3.6. DVL Error Equation

The DVL measures the Doppler frequency shift, and then provides three-dimensional velocity measurements. In general, the DVL has two modes of operation, i.e., the bottom lock mode and the bottom unlock mode. When the DVL is used to provide the velocity of the vehicle related to the seafloor, it operates in bottom lock mode. The main error sources of the DVL contain the sound velocity errors and the frequency shift biases. In the DVL output, the sound velocity errors mainly cause the scale factor errors and the frequency shift biases mainly lead to the random velocity errors. The DVL output in the DVL body frame (donated as m frame) can be presented as:

(40) $${\tilde{V}}_{DVL}^{m}=(I+\delta K)V_{DVL}^{m}+\delta V_{DVL}^{m}$$

where $V_{DVL}^{m}$ is the actual velocity in m frame; δK is the scale factor error; and $\delta V_{DVL}^{m}$ is the random velocity error.

When the DVL output is translated to navigation frame, the main DVL output errors also include the installation error ψ and the attitude error φ. The DVL output in navigation frame (i.e., G frame) can be obtained by:

(41) $${\tilde{V}}_{DVL}^{G}=[I-\varphi \times ]C_{b}^{G}[I-\psi \times ]C_{m}^{b}{\tilde{V}}_{DVL}^{m}$$

Neglecting the second order small quantity, the DVL output in G frame can be rewritten as:

(42) $${\tilde{V}}_{DVL}^{G}=C_{b}^{G}C_{m}^{b}V_{DVL}^{m}+\delta KC_{b}^{G}C_{m}^{b}V_{DVL}^{m}+C_{b}^{G}C_{m}^{b}\delta V_{DVL}^{m}+C_{b}^{G}C_{m}^{b}(V_{DVL}^{m}\times )\varphi +C_{b}^{G}C_{m}^{b}(V_{DVL}^{m}\times )\psi$$

In the practical application, due to some special marine environments, the DVL may fail to maintain bottom lock and lead to the following three consequences: firstly, the DVL output will contain noises with uncertain statistical characteristic, which are mostly large-amplitude errors and intensity mutation errors; secondly, the DVL output will provide velocities related to the ocean currents, which are shown as constant velocity errors; and, finally, no velocity information will be provided by the DVL. When the DVL is not able to provide vehicle velocities, the navigation system will change from the integrated mode to the independent inertial navigation mode. In this paper, the first two situations will be discussed. Both kinds of DVL output errors contain uncertain noises with unknown characteristics and are hard to model accurately.

## 4. Design of the Grid SINS/DVL Integrated Navigation Algorithms

The grid SINS can ensure the reliability and accuracy independently in short term. However, due to the errors of inertial measurement unit (IMU), the navigation accuracy will decrease sharply when the grid SINS works independently for a long time. To ensure the navigation accuracy, an integration of grid SINS and DVL is adopted.

The traditional grid frame integrated navigation systems have different Earth models between the mechanization and the filter model, which will bring the principle error and decrease the navigation accuracy.

Besides, the traditional integrated navigation systems are designed by the traditional KF. The KF is an optimal state estimator based on the assumption that the system dynamic model and noise statistic characteristics are known precisely. However, the KF would not be optimal when used in practice due to the uncertain measurement noises and outliers.

Finally, the traditional integrated system with the output-correction or the feedback-correction has its own shortages. The output-correction corrects the SINS output errors directly. The traditional integrated system with the output-correction can converge with a fast speed when the measurement contains mutation errors, but the SINS inner errors are not corrected and still accumulate with time. Due to the accumulated error, when working for long hours the filter state equation will not match the actual SINS working state and the filter accuracy will decrease or even diverge. The ships or submarines always work for long hours in or heading to the polar region, so the output-correction is hardly suited for the navigation task. In contrast, the feedback-correction can correct the SINS inner errors with the same update frequency of the filter, but the filter output will be involved in SINS calculation. As a result, the filter errors become one of the SINS error sources. When the measurement contains unexpected mutation errors, the feedback-correction system will converge slowly compared to the output-correction and the filter estimation results will contain serious errors. If the serious errors are introduced into the SINS, the navigation accuracy will decrease seriously and the influence will exist for a long time. In practice, the uncertain DVL output errors are unavoidable, so the feedback-correction is not a proper correction method.

To overcome the disadvantages of the traditional system discussed above and improve the navigation performance for marine applications, a novel grid SINS/DVL integrated navigation algorithm is designed with the unified Earth model and an ARKF based hybrid-correction scheme is proposed in the following sections.

### 4.1. Dynamic Model

The dynamic models of the integrated navigation system include grid SINS model and DVL model. Assuming that constant errors (denoted as $\varepsilon_{c}^{b}$ and $\nabla_{c}^{b}$) and random errors (denoted as $\varepsilon_{w}^{b}$ and $\nabla_{w}^{b}$) exist in gyroscope and accelerometer outputs, the gyroscope and accelerometer output errors can be denoted as:

(43) $$\delta {\tilde{\omega }}_{ib}^{b}=\;\varepsilon_{c}^{b}+\varepsilon_{w}^{b}$$

(44) $$\delta {\tilde{f}}^{b}=\nabla_{c}^{b}+\nabla_{w}^{b}$$

The attitude error ϕ, velocity error $\delta V^{G}$, position error in ECEF frame $\delta R^{e}$, drifts of gyroscope $\varepsilon^{b}$ and accelerometers $\nabla^{b}$ are chosen as the states of the grid SINS to be estimated, i.e., $x_{SINS}={\left[\begin{array}{ccccc} \phi & \delta V^{G} & \delta R^{e} & \varepsilon_{c}^{b} & \nabla_{c}^{b} \end{array}\right]}^{T}$. According to the derived error equations of grid SINS in Section 3, the differential equations of grid SINS states can be described as:

(45) $$\{\begin{array}{l} \dot{\phi }\;=-\omega_{iG}^{G}\times \phi +C_{V\_\phi }\delta V+C_{R\_\phi }\delta R^{e}-C_{b}^{G}\varepsilon^{b}\\ \delta {\dot{V}}^{G}\;\;\;\;=\;f^{G}\times \phi +C_{V\_V}\delta V^{G}+C_{R\_V}\delta R^{e}+C_{b}^{G}\nabla^{b}\\ \begin{array}{l} \delta {\dot{R}}^{e}\;\;=\;C_{V\_R}\delta V^{G}+C_{R\_R}\delta R^{e}\\ {\dot{\varepsilon }}_{c}^{b}\;\;\;\;\;\;\;\;=\;\;\;\;\;\;\;0 \end{array}\\ {\dot{\nabla }}_{c}^{b}\;\;\;\;\;\;=\;\;\;\;\;\;\;0 \end{array}$$

The DVL state is $x_{DVL}=\delta V_{DVL}^{m}$, and the random velocity error is assumed as the one-order Markov process:

(46) $$\delta {\dot{V}}_{DVL}^{m}=-\delta V_{DVL}^{m}/\tau_{V}+w_{V}$$

where $\tau_{V}$ is the correlation time of Markov process and $w_{V}$ is the zero-mean Gaussian white noise.

Then, the dynamic model of the integrated system can be described by state equations as follow:

(47) $$\left[\begin{array}{c} {\dot{x}}_{\mathrm{SINS}}\\ {\dot{x}}_{\mathrm{DVL}} \end{array}\right]=\left[\begin{array}{cc} F_{SINS} & 0_{15\times 3}\\ 0_{3\times 15} & F_{DVL} \end{array}\right]\left[\begin{array}{c} x_{SINS}\\ x_{DVL} \end{array}\right]+\left[\begin{array}{cc} B_{SINS} & 0_{15\times 3}\\ 0_{3\times 6} & B_{DVL} \end{array}\right]\left[\begin{array}{c} w_{SINS}\\ w_{DVL} \end{array}\right]$$

where F is the system matrix; B is the noise transition matrix; and w is the system noise. $w_{SINS}={\left[\begin{array}{cc} \varepsilon_{w}^{b} & \nabla_{w}^{b} \end{array}\right]}^{T}$, $w_{DVL}=\;w_{V}$, and

(48) $$F_{SINS}=\left[\begin{array}{ccccc} -\omega_{iG}^{G}\times & C_{V\_\phi } & C_{R\_\phi } & -C_{b}^{G} & 0_{3\times 3}\\ f^{G}\times & C_{V\_V} & C_{R\_V} & 0_{3\times 3} & C_{b}^{G}\\ 0_{3\times 3} & C_{V\_R} & C_{R\_R} & 0_{3\times 3} & 0_{3\times 3}\\ 0_{3\times 3} & 0_{3\times 3} & 0_{3\times 3} & 0_{3\times 3} & 0_{3\times 3}\\ 0_{3\times 3} & 0_{3\times 3} & 0_{3\times 3} & 0_{3\times 3} & 0_{3\times 3} \end{array}\right]$$

(49) $$B_{SINS}=\;\left[\begin{array}{cc} -C_{b}^{G} & 0_{3\times 3}\\ 0_{3\times 3} & C_{b}^{G}\\ 0_{9\times 3} & 0_{9\times 3} \end{array}\right]$$

(50) $$F_{DVL}=\;\left[\begin{array}{ccc} -1/\tau_{Ve} & 0 & 0\\ 0 & -1/\tau_{Vn} & 0\\ 0 & 0 & -1/\tau_{Vu} \end{array}\right]$$

(51) $$B_{DVL}=\;I_{3\times 3}$$

where $C_{V\_\phi }=C_{3}$, $C_{R\_\phi }=(C_{6}+C_{2})C_{R2P}+(C_{5}+C_{4})C_{R2\sigma }$, $C_{R\_v}=\;\;\;(V^{G}\times )(2C_{6}+C_{2})C_{R2P}+(V^{G}\times )(2C_{5}+C_{4})C_{R2\sigma }$, $C_{v\_v}=(V^{G}\times )C_{3}-(2\omega_{ie}^{G}+\omega_{eG}^{G})\times$, and $C_{V\_R}=C_{G}^{e}$, $C_{R\_R}=-C_{G}^{e}(V^{G}\times )C_{R2\delta \theta }$.

### 4.2. Observation Model

Both the grid SINS and DVL can output vehicle velocity. The velocity error between grid SINS and DVL is chosen as the system observation. Besides, the DVL installation error and scale factor error are compensated by calibration and neglected in this paper. According to Equation (42), the DVL output in G frame can be obtained by:

(52) $${\tilde{V}}_{DVL}^{G}=V^{G}+(V_{DVL}^{G}\times )\varphi +\delta V_{DVL}^{G}$$

where $V^{G}$ is the actual vehicle velocity in the G frame and $\delta V_{DVL}^{G}$ is the random velocity error of DVL.

The grid SINS output velocity can be described as:

(53) $${\tilde{V}}_{SINS}^{G}=V^{G}+\delta V_{SINS}^{G}$$

where $\delta V_{SINS}^{G}$ is the SINS output error in G frame.

The observation model of integrated system can be given by:

(54) $$Z={\tilde{V}}_{SINS}^{G}-{\tilde{V}}_{DVL}^{G}=Hx+\nu$$

where the observation matrix H can be described as $H=\left[\begin{array}{ccc} V_{DVL}^{G}\times & I_{3\times 3} & 0_{12\times 3} \end{array}\right]$, and ν is the system measurement noise.

### 4.3. The Adaptive Robust Kalman Filter

Because the DVL output errors with imprecise or unknown statistics are hard to be modeled, an ARKF is employed to restrain the uncertain measurement errors and improve the filter accuracy. The adaptive factor is used in the ARKF algorithm to adjust the filter algorithm. The ARKF can detect and reduce the measurement mutation random errors.

The ARKF is based on M-estimation, and the filter steps are described as the follows [28,29]:

(55) $${\hat{X}}_{k,k-1}=\Phi_{k,k-1}{\hat{X}}_{k-1}$$

(56) $$P_{k/k-1}=\Phi_{k,k-1}P_{k-1}\Phi_{{}^{{}_{k,k-1}}}^{T}+\Gamma_{k-1}Q_{k-1}\Gamma_{k-1}^{T}$$

(57) $$K_{k}=P_{k/k-1}H_{k}^{T}{\left(H_{k}P_{k/k-1}H_{k}^{T}+{\bar{R}}_{k}\right)}^{-1}$$

(58) $${\hat{X}}_{k}={\hat{X}}_{k/k-1}+K_{k}(Z_{k}-H_{k}{\hat{X}}_{k/k-1})$$

(59) $$P_{k}=\left(I-K_{k}H_{k}\right)P_{k/k-1}{\left(I-K_{k}H_{k}\right)}^{T}+K_{k}{\bar{R}}_{k}K_{k}^{T}$$

The institute of geodesy and geophysics (IGG) III function of the adaptive factor $\alpha_{i}$ is obtained by [28,29]:

(60) $$\alpha_{i}=\{\begin{array}{cc} 1 & \left|\Delta {{\bar{V}}^{i}}_{k,k-1}\right|\leq c_{0}\\ \frac{c_{0}}{\left|\Delta {{\bar{V}}^{i}}_{k,k-1}\right|}{\left(\frac{c_{1}-\left|\Delta {{\bar{V}}^{i}}_{k,k-1}\right|}{c_{1}-c_{0}}\right)}^{2} & c_{0}\leq \left|\Delta {{\bar{V}}^{i}}_{k,k-1}\right|\leq c_{1}\\ 0 & \left|\Delta {{\bar{V}}^{i}}_{k,k-1}\right|>c_{1} \end{array}$$

where $\left|\Delta {{\bar{V}}^{i}}_{k,k-1}\right|$ is the standardized predicted residual of measurement: $\left|\Delta {{\bar{V}}^{i}}_{k,k-1}\right|=\left|Z_{k}-H_{k}{\hat{X}}_{k/k-1}\right|/\sigma_{R}$; and $\sigma_{R}$ is the standard deviation of measurement noise. $\left|\Delta {\bar{V}}^{i}{}_{k,k-1}\right|$ can reflect the measurement accuracy to some extent. $c_{0}$ and $c_{1}$ are constant thresholds of $\left|\Delta {{\bar{V}}^{i}}_{k,k-1}\right|$ and $1.0<c_{0}<1.5$, $3.0<c_{1}<4.5$. When the measurement contains small errors, $\alpha_{i}=1$ and the measurement is involved in filter update with original value. When the measurement contains unpredicted measurement errors, $0<\alpha_{i}<1$ and the weight of measurement is reduced. When the measurement contains large unpredicted measurement errors, $\alpha_{i}=0$ and the measurement is rejected to participate in the filter update.

The equivalent covariance of the measurement noise is related to the DVL measurement, and the equivalent covariance ${\bar{R}}_{k}$ can be obtained by [28,29]:

(61) $${\bar{R}}_{k}=R/\alpha_{i}$$

When the ARKF is applied to the grid SINS/DVL integrated navigation system, measurement outliers will be isolated or weighted less to enhance the accuracy of navigation system.

### 4.4. The ARKF Based Hybrid-Correction Scheme

In this paper, considering the grid SINS/DVL integrated system character, an ARKF based hybrid-correction scheme is proposed to improve the navigation accuracy.

**Figure 5:**
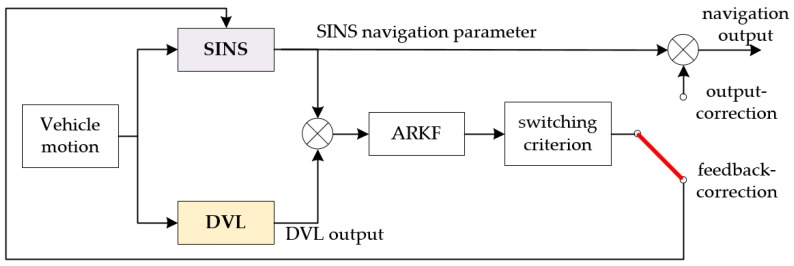
The Adaptive robust Kalman filter (ARKF) based hybrid-correction integrated navigation structure diagram.

The ARKF based hybrid-correction scheme contains three parts, i.e., an output-correction channel, a feedback-correction channel and a two-order switching criterion. The ARKF based hybrid-correction integrated navigation structure diagram is shown in Figure 5.

**Figure 6:**
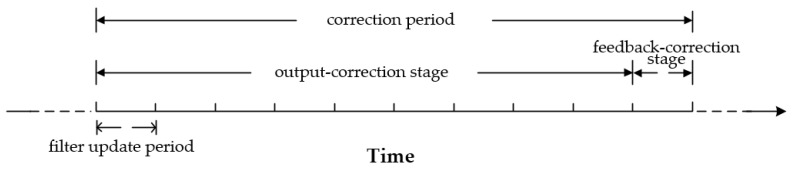
Description of the correction period.

A certain correction period is given by the empirical value. As shown in Figure 6, a correction period contains two stages, i.e., an output-correction stage and a feedback-correction stage. The output-correction stage is nine times as long as the filter update period, and is always at the beginning of every correction period. During the output-correction stage, the output-correction operates at the same frequency with the filter update. The feedback-correction stage is as long as the filter update period, and is always at the end of every correction period. Besides, the feedback-correction operates at most one time during every correction period.

**Figure 7:**
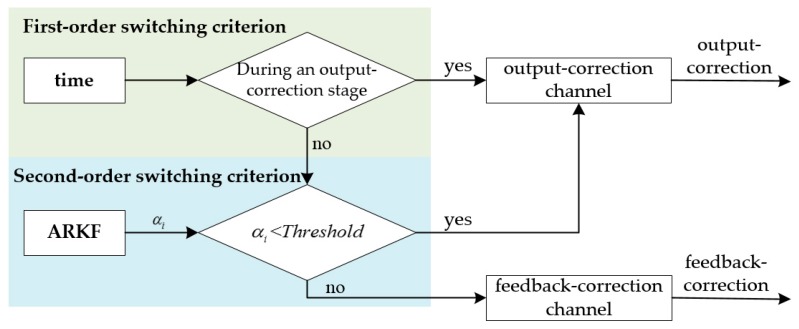
Two-order switching criterion structure diagram.

The two-order switch criterion is used to choose the output-correction channel or the feedback-correction channel, which is shown in Figure 7.

As shown in Figure 7, the first order switch criterion is a time criterion. In the output-correction stage, the output errors will be restrained by the output-correction to ensure the navigation output accuracy. Then the SINS inner errors will be corrected by the feedback-correction in the feedback-correction stage, which will ensure the filter state equations to match the actual system properly. Besides, the feedback correction frequency of the hybrid-correction scheme is lower than the filter update frequency, so the filter errors of hybrid-correction system have less influence to the grid SINS performance than the traditional feedback-correction system. Although the hybrid-correction scheme with time switch criterion is able to improve navigation performance to some extent, a part of filter errors will still be feedback into the grid SINS, especially when the DVL output errors contain large amplitude or mutation. To further resist the filter errors, the ARKF with an adaptive factor $\alpha_{i}$ is used and a second order switch criterion is designed as a threshold criterion. The ARKF adaptive factor $\alpha_{i}$ is used to detect and resist the unpredicted measurement errors. During the feedback-correction stage, if the DVL operates properly and $\alpha_{i}\geq threshold$, the correction channel will change from the output-correction channel to the feedback-correction channel to correct the grid SINS inner errors; if the DVL output measurement contains large noise and $\alpha_{i}<threshold$, the correction channel will not change from the output-correction channel to the feedback-correction channel, so that the filter errors will not be introduced into the grid SINS.

## 5. Experiment Results and Discussions

To validate the necessity and advantages of the filter model with unified Earth model, the ARKF algorithm and the ARKF based hybrid-correction scheme, the simulations and experiments are conducted in middle and high latitude regions respectively.

To estimate the performances of the designed algorithm, the sea state and vehicle motion status are also considered. The sea state is set as moderate condition, and the motion status includes static, motion with constant velocity, motion with constant acceleration, climbing and turning a corner. The main IMU and motion parameters of the grid SINS trajectory generator are described as follows.

Firstly, the gyroscopes drifts are set as 0.03° per hour and the random errors of gyroscopes are set up as zero-mean Gaussian white noises. The accelerometer biases are set as 5 × 10^−5^ and the random errors of accelerometers are set up as zero-mean Gaussian white noises.

Secondly, the attitude of the vehicle is set as a sine function to simulate the influence of the moderate sea state. The amplitude and period (denoted as amplitude/period) of pitch angle, roll angle and yaw angle are set as 1°/9  s, 2°/6  s and 3°/8  s, respectively.

Thirdly, the acceleration of the vehicle is set as $0.2\;\;m/s^{2}$ and the vehicle has the maximum speed 5  m/s. The middle latitude and longitude of the initial position $P_{1}$ is set as (45°  N,   126°  E). The high latitude and longitude of the initial position $P_{2}$ is set as (75°  N,   126°  E).

Finally, the DVL output errors are set up according to the different experiment requirements, which will be illustrated in the following sections.

The simulation experiments compare different filter models, correction schemes and filter algorithms.

### 5.1. Simulation Results and Discussions

#### 5.1.1. Filter Models

There are two integrated filer models based on two different grid SINS error equations discussed in this section. The first model assumes the Earth as a sphere and the second model proposed in this paper assumes the Earth as a reference ellipsoid. Other parameters of the two methods are as follows: the DVL output errors are set up as the zero-mean Gaussian white noises. The KF is chosen as the filter algorithm. The output-correction is chosen as correction scheme and the simulation time is set up to 8 h.

It is noted that the integrated navigation system based on the two filter models can both restrain the errors of grid SINS, but the proposed model can unify the Earth model of the SINS mechanization and the integrated filter. The simulation experiment is to compare the navigation performance of integrated filter models and choose the better one.

**Figure 8:**
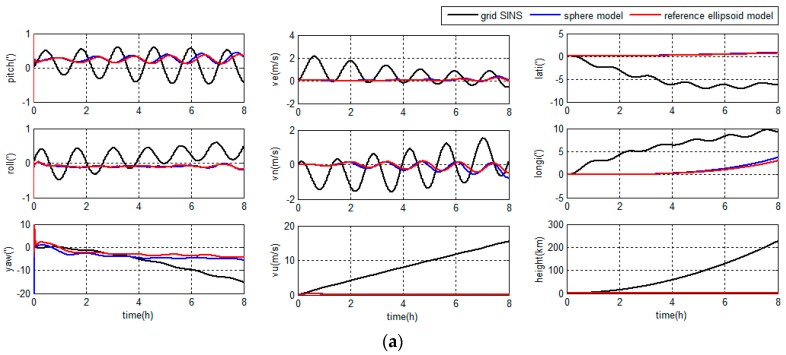

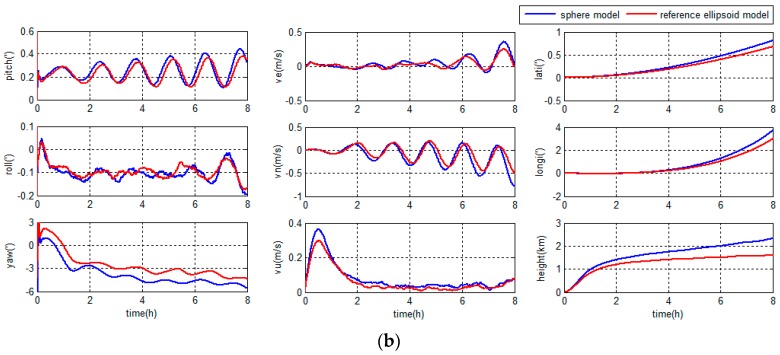
Navigation errors in middle latitude regions: (**a**) grid strapdown inertial navigation system (SINS) errors and integrated navigation errors; and (**b**) integrated navigation errors.

The navigation errors in middle latitude regions are depicted in Figure 8 and Table 1.

**Figure 9:**
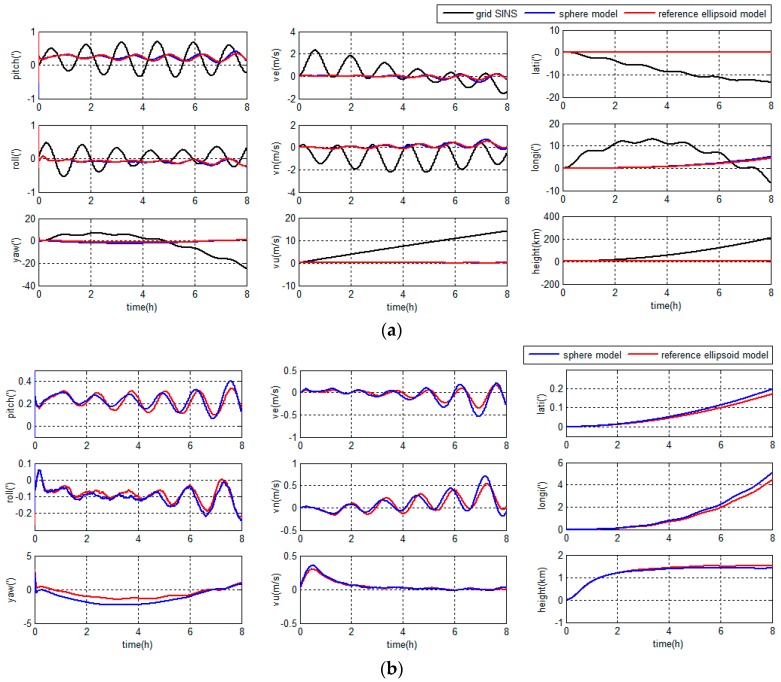
Navigation errors in high latitude regions: (**a**) grid SINS errors and integrated navigation errors; and (**b**) integrated navigation errors.

The navigation errors in high latitude regions are depicted in Figure 9 and Table 2.

As shown in Figure 8 and Figure 9, compared with the grid SINS, the oscillation and accumulated errors of the grid SINS output have been restrained by the integrated navigation algorithm. Both grid SINS/DVL integrated navigation methods have a higher accuracy than the grid SINS. Moreover, the integrated navigation system with unified reference ellipsoid Earth model has higher accuracy, less accumulated position error and smaller oscillated error amplitude, compared to the traditional integrated algorithm with sphere based filter model.

As shown in Table 1 and Table 2, when the Earth model is changed from the sphere to the reference ellipsoid, the accuracies of latitude, longitude and height are improved 16.28%, 19.21% and 14.37% (RMS), and 16.25%, 19.09% and 10.96% (maximum error), respectively, in middle latitude regions, and the accuracies of latitude, longitude and height are improved 13.20%, 12.46% and 6.22% (RMS), and 13.21%, 12.86% and 2.61% (maximum error), respectively, in high latitude regions. When choosing the ellipsoid based dynamic model, the position accuracy improves more in middle latitude regions than in high latitude regions. Because the Earth model influences the position accuracy more in middle latitude regions than in high latitude regions, and the latitude would influence the accuracy of other navigation parameters in the navigation system.

The simulation results show that the proposed reference ellipsoid based filter model can provide a better accuracy than the traditional filter model because of the unity of Earth models.

#### 5.1.2. Correction Schemes

There are three correction schemes discussed in this section: the output-correction, the feedback-correction and the hybrid-correction. All three correction schemes can be used in the grid SINS/DVL integrated navigation system to resist SINS navigation errors. The former two schemes are traditional methods and the third scheme is the one proposed in this paper. The simulation experiment is to compare the performance of the three correction schemes.

In this simulation, the DVL output errors are set up as zero-mean Gaussian white noises. The reference ellipsoid based model is chosen as the integrated filter model which has been discussed above. The KF is chosen as the filter algorithm, which means the hybrid-correction only have the first order time criterion. The simulation time is set up to 24 h.

**Figure 10:**
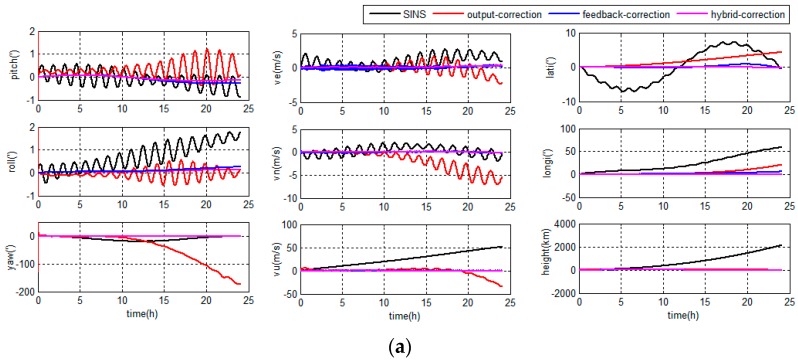

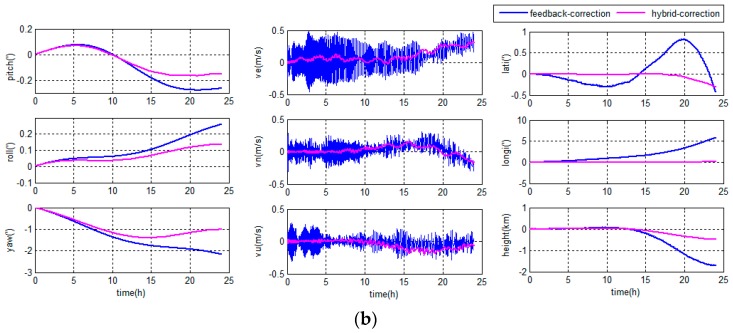
Navigation errors in middle latitude regions: (**a**) grid SINS errors and integrated navigation errors; and (**b**) feedback and hybrid correction integrated navigation errors.

The navigation errors in middle latitude regions are depicted in Figure 10.

**Figure 11:**
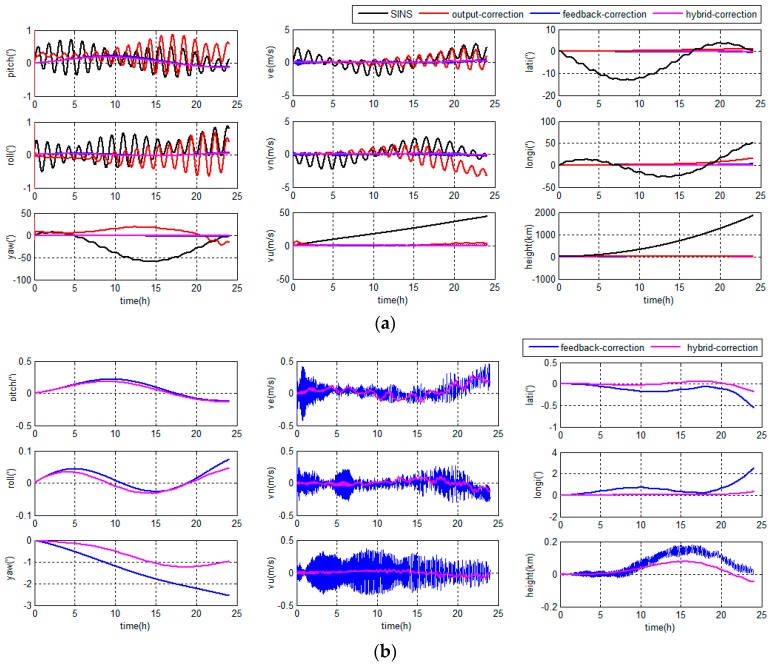
Navigation errors in high latitude regions: (**a**) grid SINS errors and integrated navigation errors; and (**b**) feedback and hybrid correction integrated navigation errors.

The navigation errors in high latitude regions are depicted in Figure 11.

As shown in Figure 10 and Figure 11, if the integrated system works less than 10 h, the output-correction system can maintain a certain accuracy. However, if the integrated system works for long hours, the output-correction system navigation errors will be accumulated and even larger than grid SINS independently, because the SINS inner errors are not corrected and the filter model does not match the actual SINS state. In contrast, the filter of the feedback-correction system would not diverge, as the SINS inner errors are corrected after the filter estimation. However, the feedback-correction system is sensitive to DVL output errors, although the DVL errors are set as zero-mean Gaussian white noises.

The navigation error curves of the hybrid-correction system are shown in Figure 10 and Figure 11. The hybrid system has an output-correction channel, so the navigation output errors are corrected. The output correction frequency is nearly the same as filter update frequency, and the navigation output accuracy is maintained in short term. Besides, the hybrid system also has a feedback-correction channel, so the inner errors of SINS are corrected periodically and the navigation system will maintain a proper accuracy even working for long hours. The feedback channel of the hybrid system operates at a lower frequency than the filter update, so the DVL output errors of the hybrid-correction system have less influence to the integrated navigation accuracy than the feedback-correction system.

Therefore, the grid SINS/DVL integrated navigation system with the hybrid-correction scheme has a higher navigation accuracy and better performance than traditional correction schemes.

#### 5.1.3. KF and ARKF Algorithms

The simulation experiments mentioned above all assume that DVL output errors are zero-mean Gaussian white noises, and the filter algorithm is the KF. However, when the DVL operates in practice, the DVL output may contain different kinds of errors, such as the normal DVL output noises, the unpredicted large random errors, the velocity of the ocean current, etc. In this case, the KF will not work in optimal state. To illustrate the filter algorithm, the DVL output errors in trajectory generators are adjusted to simulate the errors in practice.

**Figure 12:**
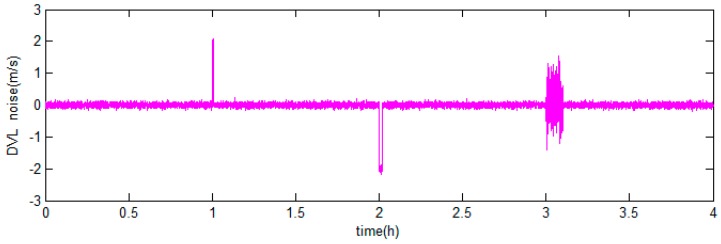
The Doppler velocity log (DVL) output errors of the simulation experiment.

There are two integrated filter algorithms discussed in this simulation experiment. The reference ellipsoid based filter model is chosen as the integrated model which has been discussed above. The filter algorithms are the KF and ARKF, respectively, so the correction schemes are the KF based hybrid-correction and the ARKF based hybrid-correction, respectively. When the DVL fails to maintain bottom lock in short time, the velocity of ocean current will be considered as a constant. The DVL output errors are shown in Figure 12.

When the DVL works properly, the errors are set up as zero-mean Gaussian white noises. When the DVL fails to maintain bottom lock during 1.0 h<t<1.01 h and 2.0 h<t<2.02 h, the constant errors 2 m/s and −2 m/s are set to simulate the situation that the DVL provides velocity relative to the ocean current. During 3.0 h<t<3.1 h, the errors with amplitudes about 1.5 m/s are set to simulate the situation that the DVL provides velocity with large random noises.

**Figure 13:**
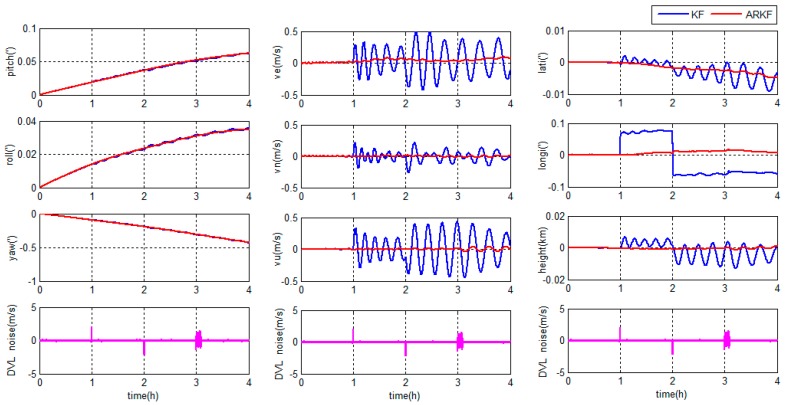
Integrated navigation errors in the middle latitude regions.

The errors of the grid SINS/DVL integrated navigation in middle latitude regions are depicted in Figure 13.

**Figure 14:**
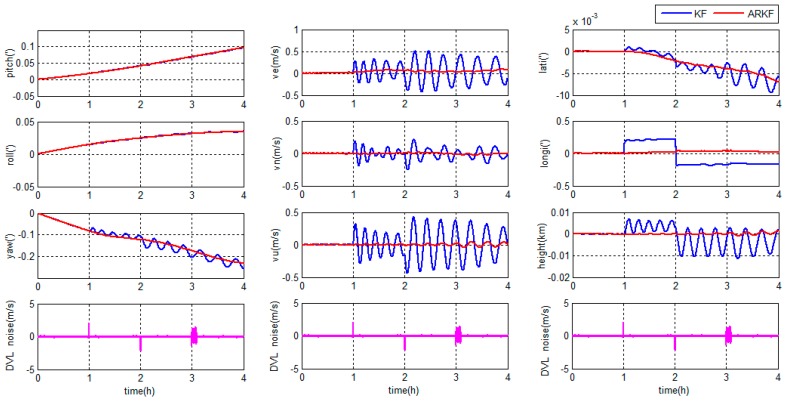
Integrated navigation errors in the high latitude region.

The errors of the grid SINS/DVL integrated navigation in high latitude regions are depicted in Figure 14.

As shown in Figure 13 and Figure 14, the hybrid-correction grid SINS/DVL integrated navigation system has different navigation performance based on different filter algorithms.

During the first one hour, the DVL works properly, the KF and ARKF have the same performance.

If the KF is chosen as the filter algorithm, large oscillation errors appear in the velocity information when the DVL fails to maintain bottom lock. At the same time, large oscillation errors and accumulated errors appear in the position information. The KF is sensitive to measurement noises and unable to restrain measurement outliers. The DVL constant errors and large amplitude random errors will increase the filter errors. When the large filter errors are introduced into the grid SINS through feedback channel, it will lead to the decrease of the navigation accuracy. Besides, the DVL constant errors have a larger influence to the navigation accuracy than large amplitude random errors.

If the ARKF is chosen as the filter algorithm, the oscillation amplitudes of velocity and position are much smaller than the KF based system and the longitude error barely accumulates with time. The reason is that the ARKF employs an adaptive factor to detect and reduce the influence of measurement uncertain errors. Meanwhile, when the adaptive factor detects a large measurement error, the switching criterion of the ARKF based hybrid-correction can reject the feedback-correction channel to avoid filter errors to be introduced into the grid SINS. Therefore, the ARKF based hybrid-correction system has a higher accuracy and stronger robustness than the traditional KF based hybrid-correction integration.

### 5.2. Semi-Physical Experiment Results and Discussions

Due to the geographic restriction of the field experiment especially the polar experiment, a semi-physical experiment is conducted in this paper. The key point of the semi-physical simulation experiment is to get the actual sensor output errors, including the IMU output errors and the DVL output errors. The actual sensor errors can reflect the actual error characteristic of the sensors. Meanwhile, the grid based SINS trajectory generator can provide the ideal sensor output data. Then, a set of semi-physical experiment sensor output data can be obtained by adding the actual sensor errors to the ideal sensor output. The semi-physical data can reflect the performance of the navigation algorithm more properly when it is used in experiments.

#### 5.2.1. The Output Error Extraction from IMU

**Figure 15:**
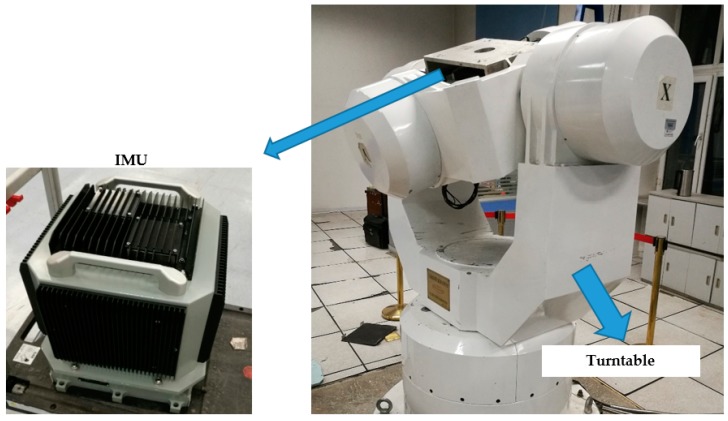
The high-precision three-axis turntable and the inertial measurement unit (IMU) in the temperature control box.

A turntable experiment is designed to extract the IMU output errors. An IMU is installed on a high-precision three-axis turntable shown in Figure 15. The main parameters of the three-axis turntable and IMU are shown in Table 3 and Table 4.

The IMU actual output data, i.e., ${\tilde{\omega }}_{ib}^{b}$ and ${\tilde{f}}_{ib}^{b}$, along with the turntable’s movement are collected. Meanwhile, the turntable’s movement data are used in the trajectory generator to output the ideal IMU measurement data, i.e., $\omega_{ib}^{b}$ and $f_{ib}^{b}$. Then, the actual output errors of the IMU can be obtained from the difference of the IMU actual output data and the ideal IMU output data.

The actual output of the gyroscope and accelerometer can be presented as:

(62) $$\left\{ \begin{array}{l} {\tilde{\omega }}_{ib}^{b}=\omega_{ib}^{b}+\delta \omega_{ib}^{b}\\ {\tilde{f}}_{ib}^{b}=f_{ib}^{b}+\delta f_{ib}^{b} \end{array}\right.$$

Then, the actual IMU output error can be obtained by:

(63) $$\left\{ \begin{array}{l} \delta \omega_{ib}^{b}={\tilde{\omega }}_{ib}^{b}-\omega_{ib}^{b}\\ \delta f_{ib}^{b}={\tilde{f}}_{ib}^{b}-f_{ib}^{b} \end{array}\right.$$

In the semi-physical integrated navigation experiment, the IMU error data $\delta \omega_{ib}^{b}$ and $\delta f_{ib}^{b}$ gained from the above turntable experiment can reflect the actual error characteristic of the gyroscopes and accelerometers. 

#### 5.2.2. The Output Error Extraction from DVL

A ship-mounted experiment is carried out in the Dalian sea area. The DVL error characteristic is susceptible to the vehicle motion status and the working environment, and a set of DVL actual output errors is extracted according to this experiment.

A high precision SINS/GNSS integrated navigation system and a DVL are mounted on a ship and both can provide ship velocity, respectively. The main parameters of the SINS/GNSS navigation system and DVL are shown in Table 5 and Table 6.

**Figure 16:**
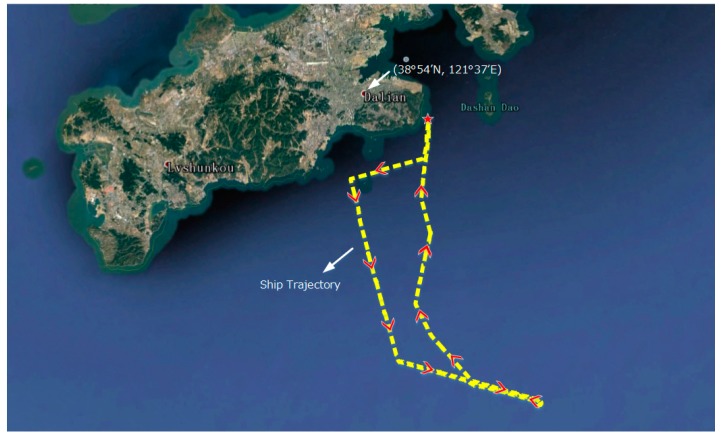
The ship trajectory of the ship-mounted experiment.

The ship trajectory in this experiment is shown in Figure 16. During the experiment, the ship has different motions, including the acceleration, deceleration, corner operation and turning tail.

The output velocity of the SINS/GNSS integrated navigation system is donated as ${\tilde{V}}_{SINS/GNSS}$ and the DVL output velocity is donated as ${\tilde{V}}_{DVL}$. After the level arm compensation and calibration, both provide the velocity of the vehicle which is expressed in the navigation frame, i.e., ${\tilde{V}}_{SINS/GNSS}^{n}$ and ${\tilde{V}}_{DVL}^{n}$. The output velocity of the SINS/GNSS integrated navigation system is much more accurate than the DVL and is considered as the reference velocity V of the actual vehicle, so the DVL output can be expressed as:

(64) $${\tilde{V}}_{DVL}^{n}=V+\delta V_{DVL}$$

Then, the DVL output errors can be calculated by:

(65) $$\delta V_{DVL}={\tilde{V}}_{DVL}^{n}-V$$

**Figure 17:**
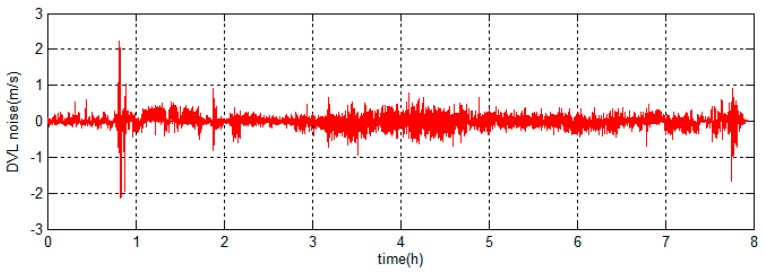
DVL output errors of the ship-mounted experiment.

The DVL output errors are shown in Figure 17.

As shown in Figure 17, the DVL output contains three kinds of errors, i.e., the noise with minor amplitude when operate properly, the noises with large amplitude and constant velocity errors when failed to maintain bottom lock.

The DVL error data $\delta V_{DVL}$ gained from the ship-mounted experiment can reflect the actual DVL error characteristic when it is used in the semi-physical integrated navigation experiment.

#### 5.2.3. Semi-Physical Grid SINS/DVL Integrated Navigation Experiments

The ideal sensor output data have been generated by the grid trajectory generator, including the gyroscopes output $\omega_{ib}^{b}$, the accelerometer output $f_{ib}^{b}$ and the DVL output V. Meanwhile, the actual noises $\delta \omega_{ib}^{b}$ $\delta f_{ib}^{b}$ and $\delta V_{DVL}$ have also been extracted from the turntable experiment and the ship-mounted experiment. Therefore, a set of semi-physical experiment sensor output data (i.e., ${\tilde{\omega }}_{ib}^{b}$ ${\tilde{f}}_{ib}^{b}$ and ${\tilde{V}}_{DVL}$) can be obtained by adding the actual sensor noises to the ideal sensor output.

There are three different kinds of grid SINS/DVL integrated navigation algorithm compared in this experiment. The former two algorithms are traditional methods. These two traditional algorithms choose the sphere as the Earth model in KF filter models, which means that the integrated system operates with different Earth models between the grid SINS mechanization and the integrated filter. The first traditional algorithm chooses the output-correction scheme and the KF, and the second traditional algorithm chooses the feedback-correction scheme and the KF. The third algorithm is the ARKF based hybrid-correction grid SINS/DVL integrated navigation algorithm proposed in this paper, which chooses the reference ellipsoid as the Earth model to unify the Earth model of the grid SINS mechanization and the integrated filter.

**Figure 18:**
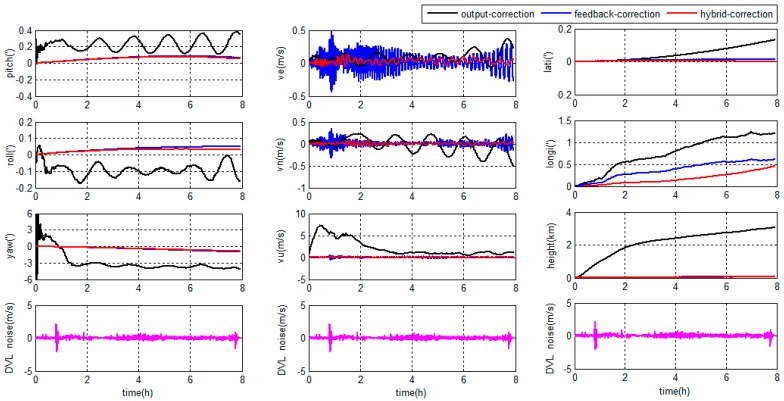
Integrated navigation errors in the middle latitude regions.

The errors of the grid SINS/DVL integrated navigation in middle latitude regions are depicted in Figure 18.

**Figure 19:**
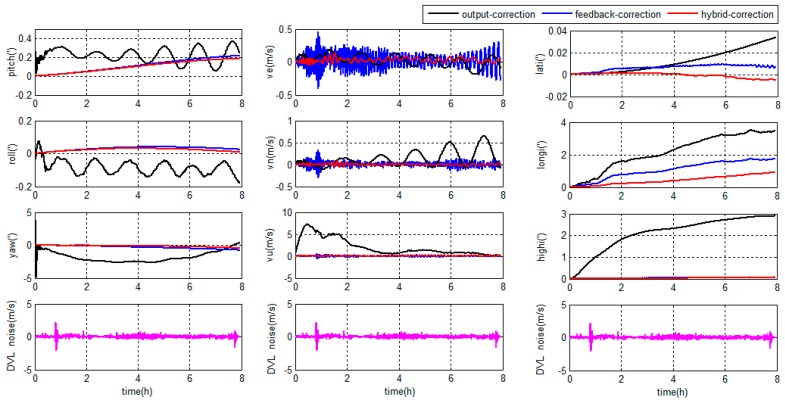
Integrated navigation errors in the high latitude regions.

The errors of grid SINS/DVL integrated navigation in high latitude regions are depicted in Figure 19.

As shown in Figure 18 and Figure 19, during the 8 h experiment, the traditional KF output-correction grid SINS/DVL integration algorithm has the lowest overall accuracy. The navigation outcomes are diverging; the Schuler oscillation is not completely suppressed; and the oscillation amplitude increases gradually. The reason is that the SINS inner errors are not corrected by the integration algorithm.

Compared with the traditional output-correction, the KF feedback-correction algorithm corrects the SINS inner errors in every filter period and the Schuler oscillation is completely suppressed. The attitude and position accuracies are higher than the output-correction system. Due to the weakness of the position observability [30], the position errors are hard to be restrained totally and still accumulate with time. Besides, the KF feedback-correction system is sensitive to the DVL output errors. When the DVL has large noises, the feedback-correction scheme even has less horizontal velocity accuracy than the output-correction scheme.

The proposed ARKF hybrid-correction system has the best performance among the three methods. Compared with the traditional KF output-correction system, the ARKF hybrid-correction system has a higher navigation accuracy. Compared with the traditional KF feedback-correction system, the proposed ARKF hybrid-correction system has the similar attitude accuracy but higher velocity and position accuracy and stronger robustness. The reasons are as follows: firstly, the unified Earth model decreases the principle errors; secondly, the navigation output errors are corrected by the output-correction channel of the hybrid-correction; thirdly, the SINS inner errors are corrected periodically by the feedback-correction channel of the hybrid-correction to ensure the navigation accuracy when working for long hours; fourthly, the ARKF filter can detect and adjust the filter algorithm to restrain the uncertain DVL output errors; and, finally, when the DVL output contains large noises, the residual filter errors after ARKF are rejected with the help of the $\alpha_{i}$ threshold. As a result, the proposed grid SINS/DVL integrated navigation algorithm has a best performance than the traditional methods for marine application.

## 6. Conclusions

A novel grid SINS/DVL integrated navigation algorithm is proposed to improve the performance of integrated navigation systems in middle-high latitude regions. The grid SINS error equations based on reference ellipsoid are firstly derived, and a more accurate integrated navigation filter model is designed with the unified Earth model to improve the integrated navigation accuracy. To resist the effects of measurement errors, the ARKF is employed and the ARKF based hybrid-correction scheme is proposed, by which the navigation performance is further improved and ensured for a long working time. The results of simulation and semi-physical experiments show that the proposed grid SINS/DVL integrated algorithm can effectively improve the navigation performance even working for long hours both in middle and high latitude regions. In the future, more kinds of external measurements will be employed to improve the integrated navigation accuracy and reliability.

## Figures and Tables

**Table 1:** Integrated navigation position errors in the middle latitude regions.

Parameters	Model	Maximum	RMS
Latitude/(′)	Sphere	0.8088	0.3710
	Reference ellipsoid	0.6774	0.3106
Longitude/(′)	Sphere	3.6926	1.2787
	Reference ellipsoid	2.9876	1.0331
Height/(km)	Sphere	1.8792	1.5127
	Reference ellipsoid	1.6732	1.2953

**Table 2:** Integrated navigation position errors in the high latitude regions.

Parameters	Model	Maximum	RMS
Latitude/(′)	Sphere	0.1968	0.0894
	Reference ellipsoid	0.1708	0.0776
Longitude/(′)	Sphere	5.0615	1.9602
	Reference ellipsoid	4.4108	1.7159
Height/(km)	Sphere	1.5175	1.3707
	Reference ellipsoid	1.4780	1.2854

**Table 3:** The main parameters of the three-axis turntable.

	Outer Axis	Middle Axis	Inner Axis	Unit
Angular position accuracy	±3/1	±3/1.5	±3/1	arc-sec
Minimum angular rate	±0.001	±0.001	±0.001	${}^{\circ }/s$
Maximum angular rate	±180	±250	±400	${}^{\circ }/s$
Angular rate accuracy and stability	5 × 10^−5^	5 × 10^−5^	5 × 10^−5^	${}^{\circ }/s$
Angular rate resolution	0.0001	0.0001	0.0001	${}^{\circ }/s$

**Table 4:** The main parameters of the IMU.

	Constant Bias	Random Bias
Gyroscope	<0.005 ${}^{\circ }/s$	<0.005 ${}^{\circ }/s$
Accelerometer	<7 × 10^−5^ g	<5 × 10^−5^ g

**Table 5:** The main parameters of the SINS/GNSS integrated navigation system.

Velocity Accuracy (m/s)		Time Accuracy	Data Rate
Horizontal	Vertical	20 ns	20 Hz
0.015	0.010		

**Table 6:** The main parameters of the DVL.

Velocity Accuracy	Velocity Range	Data Rate
±1.15% ± 0.2 cm/s	±17.0 m/s	12 Hz max
